# Supplementary material for: Ladostigil Attenuates Induced Oxidative Stress in Human Neuroblast-like SH-SY5Y Cells
Source: Biomedicines. 2021 Sep 17;9(9):1251. doi: 10.3390/biomedicines9091251 (PMC8471141; doi:10.3390/biomedicines9091251)
Supplement: Supplementary file 1 [file biomedicines-09-01251-s001.zip › biomedicines-1360915.pdf]

Supplemental Materials

Ladostigil attenuates induced oxidative stress in human neuroblast-like SH-SY5Y cells

Keren Zohar[1], Elyad Lezmi[2], Tsiona Eliyahu[1] and Michal Linial[1,*]

[1]Department of Biological Chemistry, [2]Department of Genetics, the Institute of Life Sciences, The Hebrew University of Jerusalem, Jerusalem, Israel.

Supplemental Table S1: Normalized data (by TMM) for all expressed genes >5 TMM

Supplemental Table S2: Differentially expressed genes and their statistical significance.

Supplementary Figures

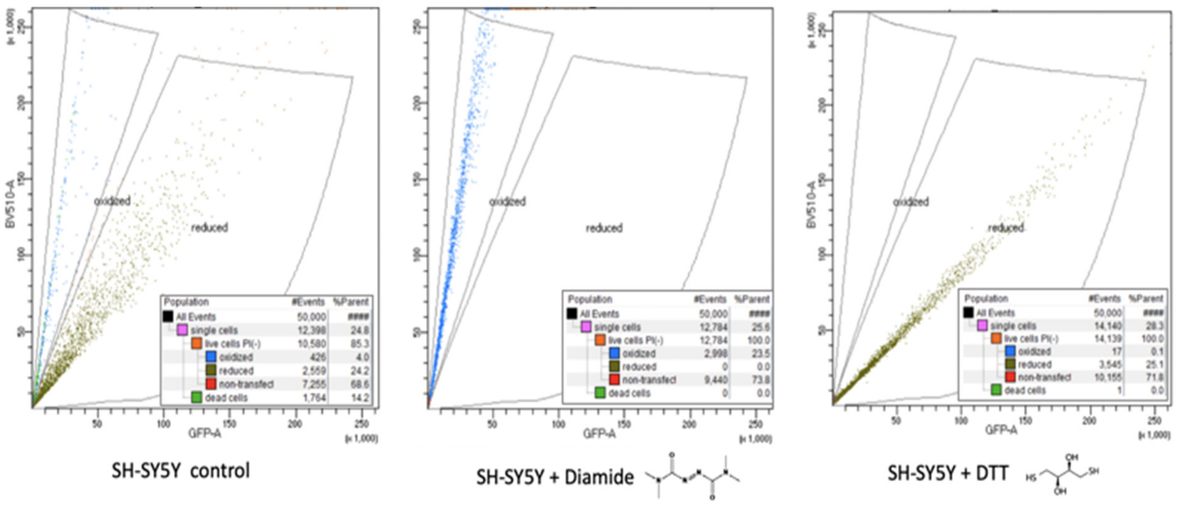

Figure S1. Calibration of redox level in cells**.** Cells were transfected with redox-measured plasmids with GFP. The transfected cells were treated after 24 hrs. The FACS gating of the extreme conditions are used as internal calibration. The FACS monitored cells after incubation with diamide which shifted all living cells toward the oxidized state (middle), and with DTT, that shifted cells to a maximal degree of reduced and oxidized states (right). The control of untreated cells (left) shows that both reduced and oxidized states can be quantified. 50,000 cells were quantified for each condition.

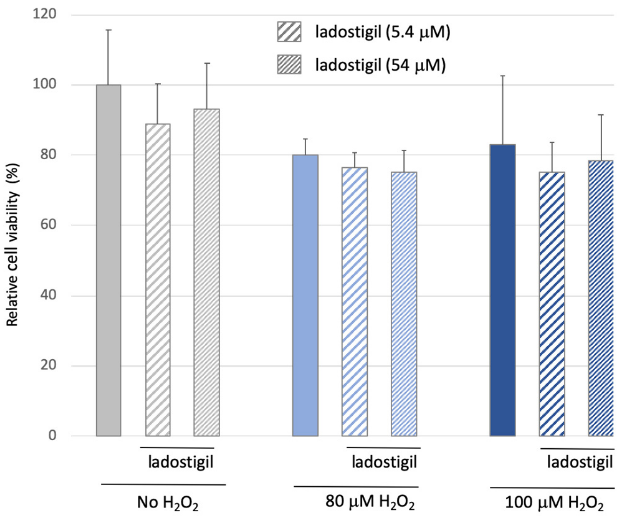

Figure S2. SH-SY5Y cell survival assay. Cells were exposed to acute oxidation stress with or without ladostigil treatment at two concentrations of ladostigil and oxygen peroxide. Each histogram bar represents the % relative to naïve untreated cells measured in 8 wells. No beneficial effect of ladostigil is measured on cell viability (24 hrs). Ladostigil was shown to be non-toxic over a wide range of concentrations.

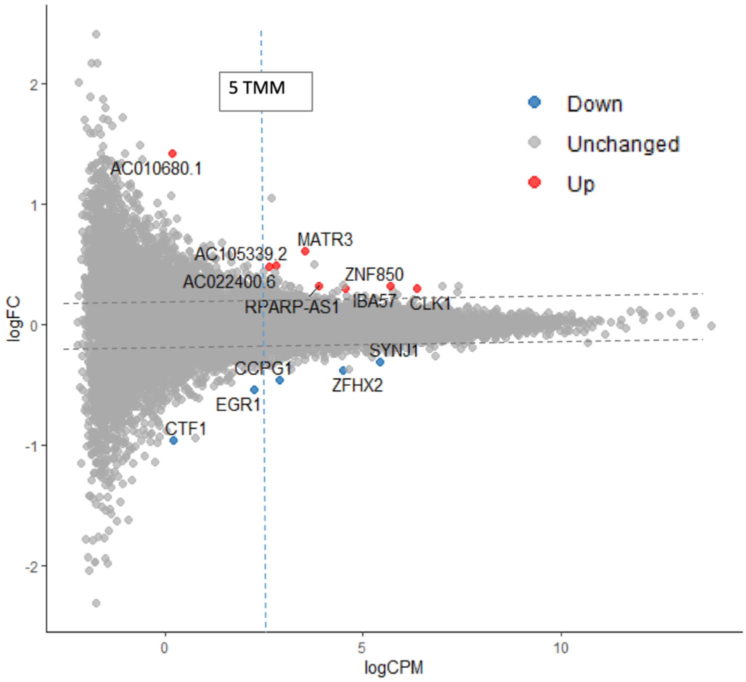

Figure S3. Differential gene expression data comparing the transcriptome of cells exposed to Sin1 (24 hrs) and cells that were pre-incubated (2 hrs) with ladostigil and then exposed to Sin1 (24 hr). Three biological replicates were used for the RNA-seq analysis. LogCPM shows the absolute expression level (TMM). The log FC (fold change) was calculated by edgeR and the horizontal lines shows the threshold for up and down regulation. The threshold of TMM=5 is marked by a dashed line.
